# A Multi-Segmented Vectoring Nozzle Configuration Inspired by the Mating Wheel of Damselfly

**DOI:** 10.3390/biomimetics11060391

**Published:** 2026-06-02

**Authors:** Bolin Liu, Linyang Chai, Chao Tian, Hengbo Chen, Huan Shen, Qian Qi, Jilei Fan, Chufei Tang, Aihong Ji

**Affiliations:** 1 Laboratory of Locomotion Bioinspiration and Intelligent Robots, College of Mechanical and Electrical Engineering, Nanjing University of Aeronautics and Astronautics, Nanjing 210014, China; sx2405022@nuaa.edu.cn (B.L.); linyang@nuaa.edu.cn (L.C.); bonnychen@nuaa.edu.cn (H.C.); shenhuan99@nuaa.edu.cn (H.S.); qiqian@nuaa.edu.cn (Q.Q.); fanjilei666@nuaa.edu.cn (J.F.); 2 Taihang National Laboratory, Chengdu 610213, China; chaotiantc@163.com; 3 Experimental Zoology Group, Wageningen University, 6708 PB Wageningen, The Netherlands; 4 Institute of Leisure Agriculture, Jiangsu Academy of Agricultural Sciences, Nanjing 210014, China

**Keywords:** *Ischnura elegans*, mating wheel, abdominal segment, morphological structure, bionic nozzle configuration, vector bending

## Abstract

Conventional thrust vector control nozzles are severely constrained by a single-pivot deflection paradigm, which induces asymmetric shock reflections and adverse boundary layer separation at large angles. Multi-segmented serial configurations offer a promising alternative to overcome these limitations by distributing the total deflection across multiple joint interfaces, thereby achieving large terminal angles and smooth flow-path curvatures. To realize such a configuration, this study draws inspiration from the abdominal bending mechanism of the damselfly *Ischnura elegans* during mating wheel formation. Real-time video recording and morphological characterizations identified abdominal segments VI and VII as critical for high-amplitude bending under load. Finite element analysis under muscular actuation elucidated the biomechanical synergy, which was rigorously verified through mesh convergence and material property sensitivity checks. Inspired by this biological system, a multi-segmented nozzle configuration incorporating discrete elastic elements and a centralized cable-driven layout was designed and evaluated using multibody dynamics and computational fluid dynamics. The nozzle achieved a continuous 61.20° deflection within 8 s under subsonic exhaust conditions, successfully stabilizing periodic supersonic shock structures and completely suppressing adverse boundary layer separation. These findings turn biological bending into a thrust vectoring method, giving insights for next-generation agile aerospace propulsion systems.

## 1. Introduction

In modern aerospace engineering, thrust vector control (TVC) is a pivotal technology that enhances a fighter’s maneuverability, post-stall agility, and stealth capabilities [1,2]. By actively adjusting nozzle geometry or exhaust flow direction, a TVC system enables complex maneuvers such as the Herbst maneuver, allowing the aircraft to execute rapid heading changes at extremely high angles of attack and achieve turn radii unattainable with aerodynamic control surfaces alone [3,4]. Additionally, thrust vectoring provides short takeoff and landing capability, enabling earlier rotation during takeoff and lower approach speeds during landing [5]. This reduces runway length requirements and increases operational flexibility in contested environments [6].

Mechanisms of conventional mechanical vectoring nozzles, however, are inherently constrained by a single-pivot deflection paradigm where the entire nozzle assembly or its convergent/divergent flaps pivot about a single point, relying on heavy-duty hinge joints [1]. Such a concentrated mechanism inherently trades off vectoring angle against mechanical interference or linkage binding. Therefore, in practice, fielded mechanical vectoring nozzles are typically limited to deflection angles of about 20° [7]. While emerging fluidic thrust vectoring technologies avoid bulky mechanical hardware by injecting secondary flows [8], they introduce substantial internal thrust losses, exhibit high sensitivity to the nozzle pressure ratio [9,10,11]. Consequently, current paradigms cannot fundamentally reconcile large deflection angles with minimal aerodynamic losses. This predicament raises a fundamental question: Does an alternative configuration paradigm exist, one distinct from single-pivot deflection, that can overcome these kinematic constraints?

From a mechanism design perspective, a multi-segmented serial configuration offers a compelling alternative. Unlike single-hinge designs that concentrate the entire deflection into a single joint, a multi-segmented architecture discretizes the total deflection angle into relative rotations across multiple sub-joints, with each joint accommodating only a fraction of the total deformation [12,13,14]. This distributed kinematic mechanism offers several distinct theoretical advantages. First, a cascaded amplification effect allows the absolute terminal deflection angle to be the cumulative sum of individual stages, thereby bypassing the physical limits inherent to single-hinge configurations [15]. Second, the flow-path curvature transitions from an abrupt single-point turn to a smooth, continuous curve; from a geometric standpoint, such a gradual transition offers the theoretical potential to mitigate severe pressure gradients, which is highly favorable for suppressing strong shock waves and delaying flow separation [16]. Furthermore, this segmented paradigm balances mechanical and actuation efficiency [17]. Third, aerodynamic bending moments and thermal stresses are distributed across multiple structural nodes, relaxing the material performance demands on any single joint [11]. Fourth, the serial mechanism, which integrates proximal actuation with distal amplification, geometrically magnifies the deflection amplitude and angular velocity of the terminal segment without requiring an increased actuator stroke, thereby providing a theoretical foundation for simplifying the internal actuator layout [18].

Notably, the literature aiming to transcend single-pivot limitations broadly falls into two categories. The first encompasses flexible continuum configurations, such as cephalopod-inspired deformable nozzles, which rely entirely on elastic material deformation [19,20]. However, simultaneously satisfying the structural stiffness, structural durability, and thermal resilience demanded by the severe exhaust environments of an aero-engine remains an immense challenge for such compliant structures [18,21]. The second category comprises rotary-jointed multi-segment configurations, typified by three-bearing swivel nozzles, which achieve deflection through rigid cylindrical segments connected by angled rotary bearings [22]. Yet, because each rotary joint demands an independent bearing and sealing system, system weight, mechanical complexity, and gas leakage risks escalate precipitously as the number of segments increases.

Existing approaches are thus constrained by a binary bottleneck: material endurance on one hand, and bearing-sealing scalability on the other [16,23,24]. This observation prompts the exploration of a third strategy, one that entrusts inter-segment relative motion to discrete elastic elements, thereby achieving a structural-functional decoupling between load-bearing and deformation at the configuration level [25]. While conceptually promising, the requisite engineering realization conditions, including high-temperature dynamic seals, coordinated multi-body control, and high-fidelity simulation tools, have historically been lacking, leaving this configuration class systematically uninvestigated in the literature [26]. Recently, however, advancements in multibody dynamics simulation and high-performance computing have made it possible to rigorously evaluate the feasibility of such configurations in a high-fidelity digital environment [27].

**Figure 1:**
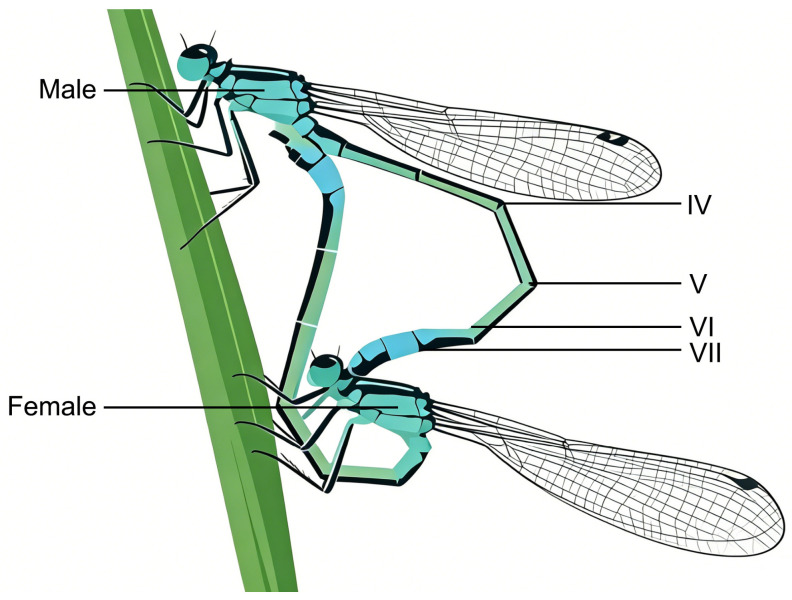
The mating wheel formed by a male and a female *Ischnura elegans*, a damselfly species, showing the abdominal bending within the wheel. Roman numerals IV to VII refer to the segment numbers.

With these digital tools in place, the challenge shifts to identifying an optimal structural blueprint. In nature, the segmented abdomen of arthropods provides an elegant multi-segment bending strategy, achieving high-amplitude flexion through the serial articulation of rigid body segments rather than continuous material compliance [28]. This strategy neatly sidesteps the thermal-mechanical vulnerabilities of flexible continua [21,29], while meeting the structural stiffness and load-carrying requirements of aerospace nozzles. Among insects, abdominal flexion is widely utilized for flight control [30]; for instance, the hawkmoth adjusts its abdominal posture to assist pitch and roll maneuvers [31]. One of the most famous cases of abdominal bending among insects occurs during the mating wheel formation of Odonata (dragonflies and damselflies) [32]. To copulate, the male bends his abdomen ventrally to achieve structural engagement with the female, forming a characteristic heart-shaped wheel [33]. In males, this high-curvature flexion is localized within abdominal segments IV to VII (Figure 1), a posture maintained under load for durations spanning minutes to several hours, often during airborne flight. Remarkably, the male damselfly can sustain flight loads reaching up to 157% of his own body weight while carrying the female [34]. This capacity to reconcile stable, large-amplitude deflection with high external load bearing aligns perfectly with the functional demands of thrust vectoring nozzles, positioning the damselfly abdomen as a compelling morphological prototype for configuration-driven nozzle innovation.

Despite its engineering potential, the exact structural mechanics enabling the damselfly’s high-curvature mating posture have remained systematically unexplored. While qualitative studies have noted the involvement of the body wall, muscles, and intersegmental membranes [22], the specific morphological adaptations, stress-handling strategies, and synergistic mechanics under load have not been quantitatively identified. More importantly, the translational feasibility of this segmented, synergistically actuated configuration into a practical aerospace nozzle remains an open question. To bridge this gap, this study progresses from biological biomechanical analysis to bio-inspired engineering design, focusing on the configuration-level innovation embodied by the damselfly abdomen. We pursue the following objectives:

(1) Kinematic Feature Extraction: Using real-time video recording, we capture the mating-wheel formation and identify the abdominal segments most critical to achieving large-angle bending by analyzing their kinematic features.
(2) Structural Characterization: Utilizing dissecting microscopy, scanning electron microscopy (SEM), confocal laser scanning microscopy (CLSM), and micro-computed tomography (Micro-CT), we elucidate the multi-scale structural differences between the curved and straight states of these critical segments, thereby identifying the core components enabling high-curvature flexibility.
(3) Mechanical Validation and Grid/Material Verification: The extracted biological features are distilled into refined mathematical models. FEA is deployed to determine stress distributions and deformation laws under muscular actuation. The structural numerical model is rigorously validated through mesh convergence and material property sensitivity checks to establish its mathematical reliability and repeatability.
(4) Bio-inspired Implementation and Aerodynamic CFD Verification: Based on the derived configuration principles, a novel multi-segmented nozzle architecture utilizing discrete elastic elements is designed. Its mechanical reliability and vector range are evaluated via multibody dynamics simulation. Furthermore, to demonstrate its practical engineering viability, two-dimensional CFD analyses were performed at deflection angles of 0°, 30°, and 60° to characterize the internal flow behavior and evaluate the thrust vectoring performance of the proposed nozzle.

## 2. Materials and Methods

### 2.1. Insects

Male specimens of the damselfly *I. elegans* were used in this study. Individuals, measuring 22–25 mm in body length, were collected in July 2025 from the Baohua Mountain population in Zhenjiang, Jiangsu Province, China. To preserve structural integrity, all specimens were stored in 70% ethanol at 4 °C.

### 2.2. Methods

#### 2.2.1. Identification of Critical Segments in Abdominal Bending Through Kinematic Analysis from Real-Time Video Recording

To identify the main abdominal segments involved in the substantial abdominal bending of damselflies, a motion capture analysis was conducted on *I. elegans* using a publicly available video titled “True Facts: Killer Carnivorous Dragon-flies” (https://youtu.be/wFAR3WggSRk?si=0B1AoV96ZJOD_5s6, accessed on 12 November 2025). The original video (30 fps, 720 p) was first enhanced to 1080 p using Video2X (v6.4.0), then frame-interpolated to 300 fps with the RIFE (Real-time Intermediate Flow Estimation) v4.26 model to meet the spatial and temporal requirements for motion capture [35].

Relative coordinates (pixel-based) of joint points between abdominal segments IV–VII were extracted frame by frame using DLTdv8 [36]. A two-dimensional coordinate system was defined based on the initial frame, following the software’s default settings (origin at upper left; *x*-axis rightward; *y*-axis downward). Pixel units were converted to real-world coordinates using a reference body length of 23.5 mm (the average size of a male of the species). A Gaussian filter was applied to coordinate data to reduce digitization noise and tracking errors. Kinematic parameters (displacement, velocity, and acceleration) were then calculated using Python (v3.12.13). Displacement was derived from Euclidean distance; velocity and acceleration were estimated using the central difference method to enhance the accuracy of instantaneous motion measurements.

Based on the above analysis, the two segments at the junction point exhibiting the greatest displacement, velocity, and acceleration were identified as the critical body segments requiring focused attention in subsequent morphological and biomechanical studies.

#### 2.2.2. Identification of Key Structures in Abdominal Bending via Multi-Scale Morphological Characterization

The structural differences between the moving (bent) and static (straight) states of the critical abdominal segments were investigated to identify the key structures enabling abdominal bending in *I. elegans*.

Specimens were preserved in 70% ethanol for stereoscopic (SteREO Discovery V.8, Carl Zeiss AG, Oberkochen, Germany) and CLSM (UltraVIEW VOX, PerkinElmer Inc., Waltham, MA, USA) to distinguish material differences between the integument and intersegmental membrane. During observation, samples were kept moist by immersion in glycerol. CLSM was performed using single-channel green light excitation.

For SEM and Micro-CT that were used to quantify morphological differences between the active (bent) and quiescent (straight) states of the abdominal segments, samples were fixed in 2.5% glutaraldehyde for 48 h, dehydrated through a graded ethanol series (70%, 75%, 80%, 85%, 90%, 95%, 100%), and dried using a critical point dryer (EM CPD300, Leica Microsystems, Wetzlar, Germany).

SEM observation (EVO-LS10, Carl Zeiss AG, Oberkochen, Germany) was employed to visualize surface cuticular microstructures, muscle fiber morphology, and integument connection points following gold-sputtering (5–15 kV). All relevant morphological parameters were measured using ImageJ 1.8.0 [37].

Three-dimensional (3D) reconstructions were performed using Micro-CT data (Xradia 620 Versa, Carl Zeiss AG, Pleasanton, CA, USA; 70 kV, 0.393× magnification). Raw 16-bit images were processed in Dragonfly 3D World 2024 (Comet Technologies Canada Inc., Montreal, QC, Canada). To ensure structural integrity, a Gaussian filter (σ = 1.2) was applied for noise reduction and artifact removal. Manual segmentation and the Poisson reconstruction algorithm were utilized to create high-resolution meshes. All relevant morphological parameters were measured using Dragonfly’s built-in tools, calibrated to a voxel resolution of 6.10 μm.

Structures showing distinct differences between the active (bent) and static (straight) states under CLSM, SEM, and 3D modeling were identified as the key components enabling damselfly abdomen bending. To ensure statistical reliability, five replicates from five individual specimens were measured for each parameter.

#### 2.2.3. Biomechanical Validation of the Key Structure’s Role in Abdominal Bending via FEA

FEA was performed in ANSYS Workbench 2024 (ANSYS Inc., Canonsburg, PA, USA) to numerically simulate the deformation process of the key components during abdominal bending. The analysis evaluated total deformation, muscle tissue deformation, and stress distribution under the maximum bending condition to assess the mechanical consistency and structural stability of the biological motion pattern, thereby clarifying the functional roles of the components.

**Figure 2:**
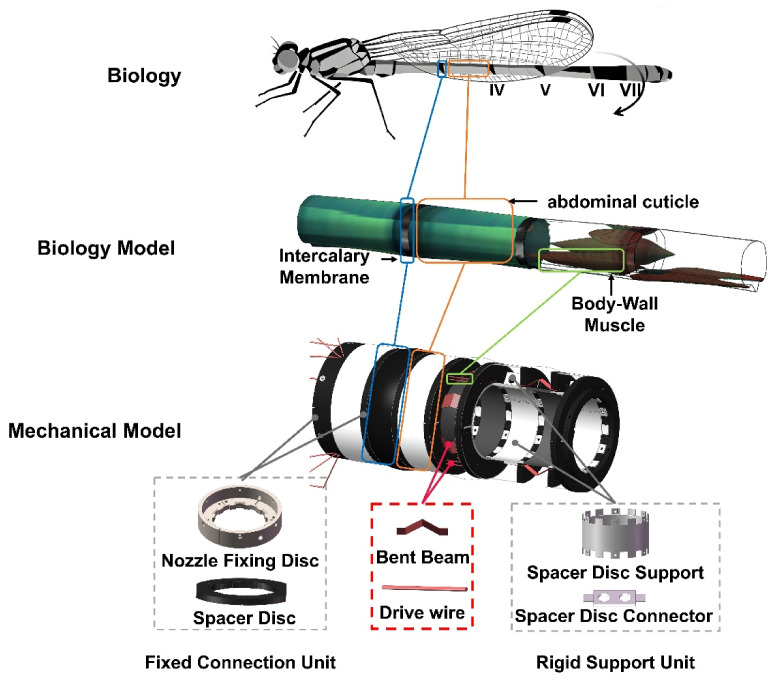
Schematic showing the progression from the biological prototype (abdomen of *I. elegans*) to its geometric model and the corresponding bio-inspired mechanical model. **Top**: biological prototype. **Middle**: geometric model for finite element analysis. **Bottom**: bio-inspired mechanical nozzle model, mapping biological structures to engineering units: rigid cuticle to rigid support unit, intersegmental membrane to flexible deformation unit, muscles to actuation system, and segment joints to fixed connection unit, realizing controllable flexible bending. Arrows indicate the mapping between corresponding components.

The geometric model (Figure 2) consisted of three components: the body wall, the muscle, and the intersegmental membrane. Their morphological traits were determined based on morphological characterization (see Section 3.2 To focus on the functional principle arising from the geometric configuration, material properties were assigned based on representative values reported in the literature for the corresponding categories of biological materials, rather than on the precise properties of a specific biological instance. The abdominal body wall was simplified as a transversely isotropic material to reflect its direction-dependent mechanical behavior, with an elastic modulus ranging from 3.0 GPa and a Poisson’s ratio of 0.3, consistent with reported values for sclerotized insect cuticle [38]. The muscle tissue was simplified as a linear elastic, nearly incompressible material (E = 0.78 MPa, ν = 0.49) to represent its contractile traction behavior, with the elastic modulus consistent with the approximately 0.3 MPa measured in maximally activated *Drosophila* flight muscles [39]. The intersegmental membrane was modeled as a low-modulus, nearly incompressible material (E = 1.0 MPa, ν = 0.49) to capture its high compliance and load-transmitting function, based on reported mechanical properties of resilin-rich biological tissues [40].

Muscle serves as the active force source driving the damselfly’s bending motion, yet reported values for its elastic modulus vary considerably in the literature [41]. Meanwhile, the body wall primarily governs the overall displacement magnitude, and the intersegmental membrane exhibits high compliance during load transmission. Therefore, a sensitivity analysis of the muscle’s elastic modulus was performed. In addition to the baseline value of 0.78 MPa, alternative values of 0.54 MPa and 1.03 MPa, representing a variation range of approximately ±32%, were evaluated to examine the impact of this parameter on the simulation results.

Regarding boundary conditions, a simplified yet mechanically explicit configuration was implemented to isolate and analyze the core mechanical response of the key segment complex, featuring a fixed anterior end and a kinematically driven posterior segment. A fixed constraint was applied to the anterior connecting cross-section to simulate the structural support from preceding body segments. Muscle contraction was then modeled by applying equivalent internal loads at the attachment sites. This simplified loading approach isolates the static force-transmission mechanism, thereby testing the structure’s inherent capacity to convert a generic contractile input into a specific bending deformation.

#### 2.2.4. Bionic Mechanical Configuration Design and Its Feasibility Evaluation via Multibody Dynamics Simulation

The design of the nozzle mechanical model (Figure 2) was based on the morphological and functional characteristics of the key structures. The body wall was mapped to segmented cylindrical shells made of metal or composite materials, comprising nozzle fixing discs, spacer discs, and spacer disc connectors. The intersegmental membrane was mapped to bending beams and nozzle sealing components, which together formed a corrugated or pleated flexible connection structure that allowed relative motion between adjacent nozzle segments while maintaining sealing integrity during movement. The muscles were mapped to a three-bundle wire-rope actuation system, which was embedded within the segmented shells (serving as the body wall mapping structures) and distributed along the longitudinal axis. By tightening and loosening the wire ropes, the contraction and relaxation effects of biological muscles were simulated, enabling control over the nozzle posture, deformation, and internal cross-sectional variation.

To evaluate the dynamic feasibility and deformation characteristics of the mechanical model, modal analysis was first performed on the bending beam in ANSYS Workbench 2024, and the corresponding Modal Neutral File (MNF) flexible body file was generated. Subsequently, a multibody dynamics simulation model was established in ADAMS 2020 (MSC Software, Newport Beach, CA, USA): the 3D nozzle model was imported into ADAMS, and the original rigid bending beam was replaced with the flexible body using the MNF file, thereby preserving the mechanical characteristics of the bending beam as a key deformation component. In the model, the two ends of the bending beam were connected to adjacent nozzle segments via fixed joints to ensure no additional relative sliding, consistent with the biological mechanism in which the intersegmental membrane is firmly attached to the adjacent body wall.

Because direct modeling of the rope drive system is complex and the focus of this study is to verify whether the configuration can achieve the intended bending mode, an equivalent simplification was adopted for the actuation method: a number of drive points equal to the number of drive ropes were placed in each layer of the active driving section, corresponding to the drive ropes in the physical structure. By applying different velocity inputs to these drive points, the tightening process of the ropes was equivalently represented as the traction effect on each nozzle segment. This approach preserves the directional traction characteristics resulting from the asymmetric contraction of biological muscle bundles while effectively avoiding the influence of secondary factors such as contact, friction, and rope path on solution efficiency.

#### 2.2.5. Fluid Dynamics and Aerodynamic Simulation Methods

To evaluate the internal flow behavior and thrust vectoring capabilities of the proposed damselfly-inspired multi-segmented nozzle, two-dimensional viscous flow field simulations were performed using the commercial software ANSYS Fluent 2025. As a fundamental mechanism study, the nozzle flow path was simplified into a rectangular domain without considering cross-sectional area contraction. The working medium was modeled as an ideal gas, with its temperature-dependent dynamic viscosity governed by Sutherland’s law.

The turbulence characteristics of the compressible high-speed flow were captured using the SST k − ω model, coupled with standard wall functions applied at the adiabatic, no-slip solid wall boundaries. The near-wall mesh height was strictly controlled at 0.001 m to resolve boundary layer characteristics accurately. Regarding boundary conditions, a mass flow inlet condition was applied at the nozzle entry, while the ambient computational domain was configured as a pressure far-field boundary with a freestream Mach number of 0.6. Numerical convergence was achieved using a density-based implicit coupled algorithm. Solution convergence was established when all tracking residuals dropped by at least three orders of magnitude (<10^−3^) and the net mass flow rate discrepancy between the inlet and outlet boundaries fell below 0.1%.

To eliminate numerical errors arising from spatial discretization, a grid independence study was systematically conducted at the initial 0° deflection state. Three distinct mesh resolutions were evaluated: a coarse mesh (14,400 elements), a medium mesh (54,800 elements), and a fine mesh (214,100 elements). The static pressure distribution along the upper wall of the nozzle was extracted as the benchmark metric. The maximum deviation rate at the peak variance point across all three grid densities was consistently less than 1%, demonstrating excellent grid convergence. Balancing computational efficiency and numerical resolution, the fine mesh model (214,100 elements) was selected for all subsequent aerodynamic vectoring calculations.

## 3. Results

### 3.1. Abdominal Segments VI and VII Are Critical for the Vectoring in Forming Mating Wheel

**Figure 3:**
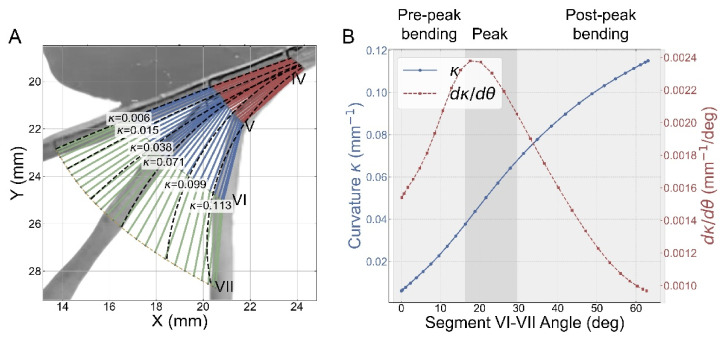
Trajectories of abdominal segments of the damselfly: (**A**) Overlay of abdominal segments (every 10 frames) and XY-plane displacement trajectories of abdominal segments IV–VII, with dashed lines indicating motion boundaries and colored regions representing movement envelopes. κ represents the curvature of an arc fitted to the actual trajectories of the abdominal segments. (**B**) Curvature (κ) variation diagram of the fitted abdomen curve. The blue solid line shows curvature as a function of angle, and the red dashed line shows its rate of change.

The motion trajectories and kinematic curves of markers on abdominal segments IV to VII, extracted from frame-interpolated video footage, systematically reveal the kinematic patterns of damselfly abdominal bending (Figure 3A). The trajectory overlay plot demonstrates that the movement of each segment can be fitted by smooth circular arcs with approximately constant curvature. During the bending process, the global abdominal curvature (denoted by κ in Figure 3A) varies from 0.006 to 0.113 mm^−1^. All markers exhibit highly synchronous temporal motion, reflecting strong global coordination across the abdominal segments. The displacement magnitude decreases sequentially from posterior to anterior along the abdominal axis (VII > VI > V > IV). This distribution aligns with the geometric characteristics of fixed-axis bending about an anterior base point, where segments closer to the distal end possess longer moment arms from the rotational center, naturally resulting in larger linear displacements. Notably, the terminal segment VII achieves the maximum cumulative displacement of approximately 10 mm.

Based on the variation in curvature, we divide the bending process into three distinct stages (Figure 3B). The first stage is pre-peak bending, where the bending angle begins to increase and the curvature rises rapidly, with κ increasing from 0.006 to 0.038 mm^−1^. The second stage is the peak acceleration stage, characterized by the maximum rate of curvature change, during which κ rises from 0.038 to 0.056 mm^−1^. The third stage is post-peak bending, where the curvature accumulation slows down markedly, with κ gradually rising from 0.056 to 0.113 mm^−1^. In brief, the rate of curvature first increases rapidly and then gradually slows down, eventually tending to a plateau. This overall kinematic phenomenon indicates a continuous, compliant deformation rather than a rigid body rotation, which is a classic characteristic of constant-curvature bio-inspired bending.

**Figure 4:**
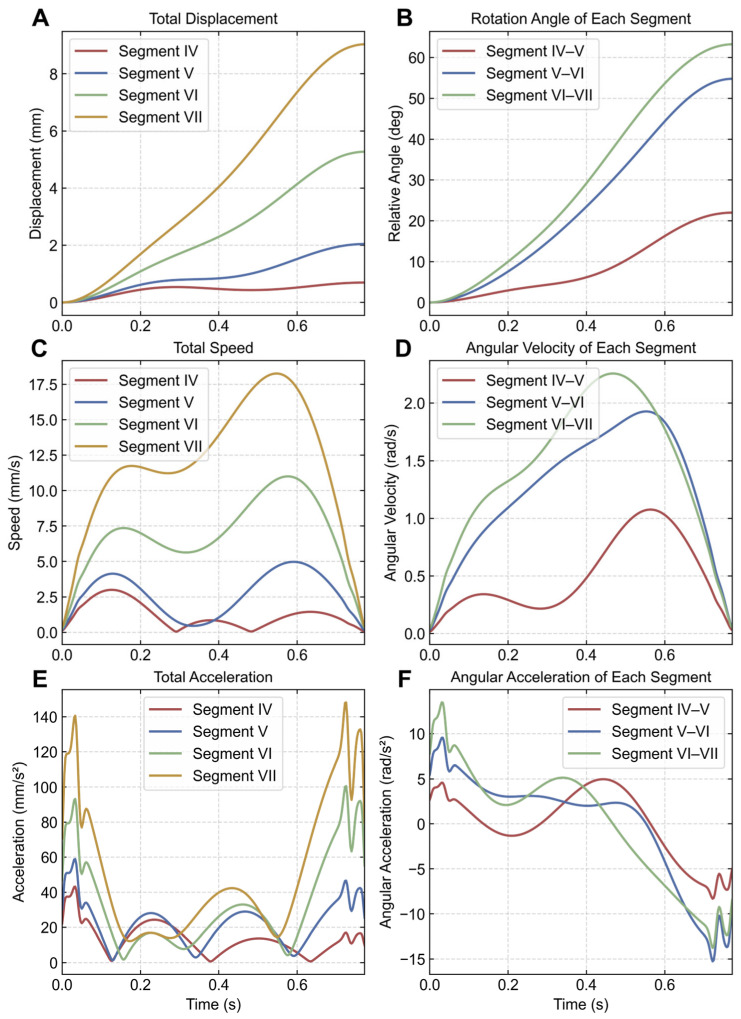
Kinematic profiles of markers on the abdominal segments of the damselfly: (**A**) Total displacement, (**B**) rotation angle of each segment, (**C**) total speed, (**D**) angular velocity, (**E**) total acceleration curves of individual abdominal segments, (**F**) angular acceleration curves of each segment.

Quantitative analysis reveals that the maximum displacement of segment VII is 70–80% greater than that of segment VI, and segment VI exhibits a comparable incremental increase relative to segment V (Figure 4A). The periodic sharp peaks in the velocity curves are highly consistent with the dense, jumping characteristics of the trajectories, while the overall motion process remains smooth, indicating that the damselfly abdomen adopts a pulsed motion mode characterized by alternating movement and stationary phases (Figure 4C). Although the abdomen can achieve high-frequency and high-speed bending, the fitted black dashed lines in Figure 3A demonstrate that the overall motion trajectory follows an approximately constant curvature characteristic. Combined with the pulsed motion regulation, this effectively ensures the smoothness of the movement.

Further analysis of the relative rotation angle (Figure 4B) and angular velocity (Figure 4D) reveals that the flexibility and dynamic actuation of the damselfly abdominal inter-segments exhibit a distinct gradient distribution: from the proximal IV–V joint to the distal VI–VII joint, the maximum relative rotation angle increases from 22° to 65°, and the peak angular velocity rises from 1.1 rad/s to 2.3 rad/s. These two quantities are highly coupled, fully validating the series flexible motion mechanism of proximal actuation and distal amplification.

Meanwhile, the smooth and continuous variation in the displacement and rotation angle curves effectively reduces the impact load exerted on the body structure by the abdominal tip motion, achieving a natural balance between high motion precision and low structural stress.

The time histories of velocity and translational acceleration also exhibit a consistent gradient distribution (Figure 4C,E). Segment VII achieves the highest peak velocity and translational acceleration, with its peak velocity being 15–25% higher than that of segment VI, accompanied by steeper acceleration peaks and more intense dynamic responses. In contrast, the anterior segments (IV and V) show smaller motion amplitudes and gentler temporal variations, further confirming that abdominal bending is mainly driven and stabilized by the anterior segments, with deformation propagating sequentially posteriorly and amplified geometrically in a stepwise manner. Further analysis of intersegmental angular acceleration (Figure 4F) corroborates this actuation mechanism: the initial impact peaks of angular acceleration decrease from the proximal to the distal abdomen, identifying the proximal intersegments as the primary actuation source. Motion is transmitted progressively through the serial multi-body structure toward the distal end, ultimately achieving large-stroke, high-dynamic output at the abdominal tip.

Based on the above analysis, abdominal segments VI and VII play a key role in the posture adjustment during the formation of the damselfly mating wheel. Their structural and kinematic characteristics of proximal actuation and distal amplification provide an important biological prototype reference for the structural design, trajectory planning, and control strategy of high-load, large-deflection flexible bending devices.

### 3.2. Muscles and Intersegmental Membrane Are Potentially Key Structure for Abdominal Bending

**Figure 5:**
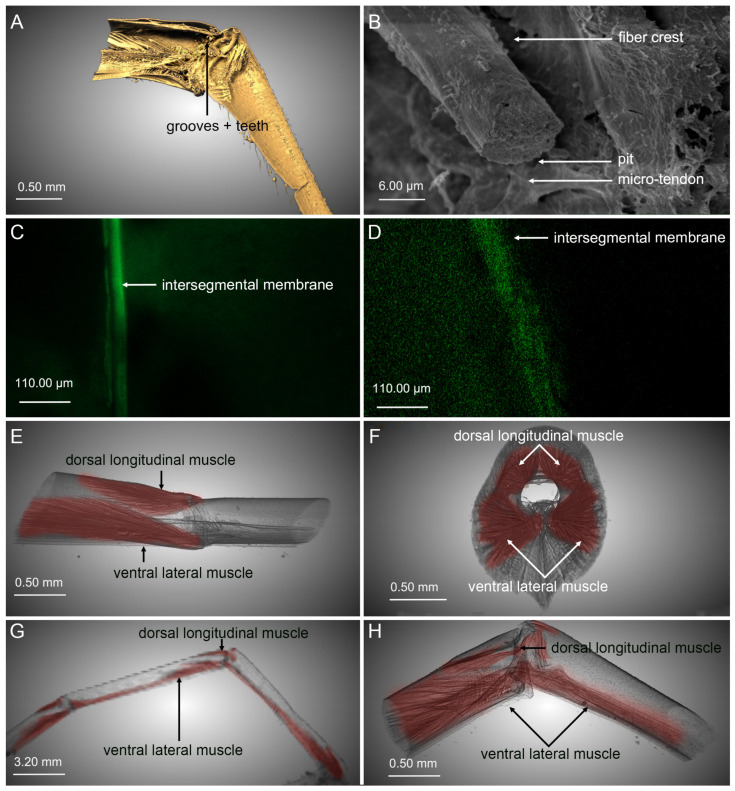
Morphology of abdominal segments VI–VII in male *I. elegans* revealed by integrated imaging. (**A**) Micro-CT reconstruction showing the interlocking articulation between segments. (**B**) SEM image detailing the attachment of muscle fibers to the integument. (**C**,**D**) CLSM visualization of the intersegmental membrane in straight (**C**) and bent (**D**) states; green fluorescence indicates the distribution of resilin-like elastic proteins. (**E**,**F**) Micro-CT reconstructions of the abdominal segments in straight state: lateral view (**E**) and posterior view (**F**). (**G**,**H**) Micro-CT reconstructions of the abdominal segments in bent state: lateral overview (**G**) and close-up of the articular region (**H**). Muscles are pseudo-colored in red in panels (**E**–**H**).

In male *I. elegans*, abdominal segments VI and VII exhibit a slender cylindrical geometry, with a length of 3.5 ± 0.5 mm and a diameter of 1.35 ± 0.15 mm. The exoskeleton forms a nested interlocking structure: the apical integument of segment VI and the basal integument of segment VII feature complementary chimeric grooves and teeth (Figure 5A). This nested configuration provides a stable physical anchor that prevents axial detachment while permitting controlled angular deflection. All muscles anchor to the integument via fiber crests, pits, and micro-tendon processes, ensuring efficient force transmission (Figure 5B). Fluorescence from CLSM tests revealed that, in contrast to the highly chitinized sclerotized integument (black), the intervening intersegmental membrane (green) is rich in resilin-like elastic proteins (Figure 5C), serving as a flexible buffer that accommodates large-scale deformation. During abdominal bending, the width of the intersegmental membrane increases to 1.8 ± 0.4 times of its original value (Figure 5D). The internal musculature consists entirely of long, fibrous, parallel-aligned muscle bundles, characteristic of typical skeletal muscle. It is functionally divided into intersegmental muscles, which drive articulation, and segmental muscles, which maintain segmental integrity (Figure 5E–H).

The intersegmental muscles are paired dorsal longitudinal muscles, which originate from the basal five-sixths of the tergum of the preceding segment and extend to the basal one-eighth of the tergum of the succeeding segment (Figure 5E). In the straight state, these muscles gradually increase in thickness from their origin to the apical one-third of their length and then decrease. At this stage, their maximum thickness reaches approximately half of the segment diameter (Figure 5F). In the bent state, these muscles become taut, shift toward the body wall, and exhibit a slight decrease in thickness (Figure 5G,H).

Segmental muscles consist of paired ventral lateral muscles, which extend diagonally from the base of the sternum to the apex of the lateral tergum and contain three bundles per side (Figure 5F,G). Their thickness increases notably starting at a point one-third of the segment length from the posterior end, reaching a maximum at one-sixth of the segment length from the posterior end, and then gradually decreases. The maximum thickness of these muscles reaches approximately two-thirds of the segment diameter (Figure 5G). When the segment bends, the thickest region of the dorsal longitudinal muscle shifts to a point approximately two-fifths of the segment length from the posterior end, and the muscle bundles tighten toward the dorsal side of the abdomen (Figure 5G,H).

From the straight state to the curved state of the abdominal segments, major morphological differences can be observed in the muscles and intersegmental membrane. Notably, all deformations are achieved with the cuticle abdominal wall as the supporting structure. Therefore, we hypothesize that the rigid body wall, the flexible and elastic intersegmental membrane, and the muscles spanning adjacent segments and connecting the tergum and sternum within each segment are the key structures. The morphology of the muscles and their attachment sites on the abdominal segments may play an important role in enabling large-angle bending.

### 3.3. Mechanical Response of Key Structures During the Abdominal Bending

**Figure 6:**
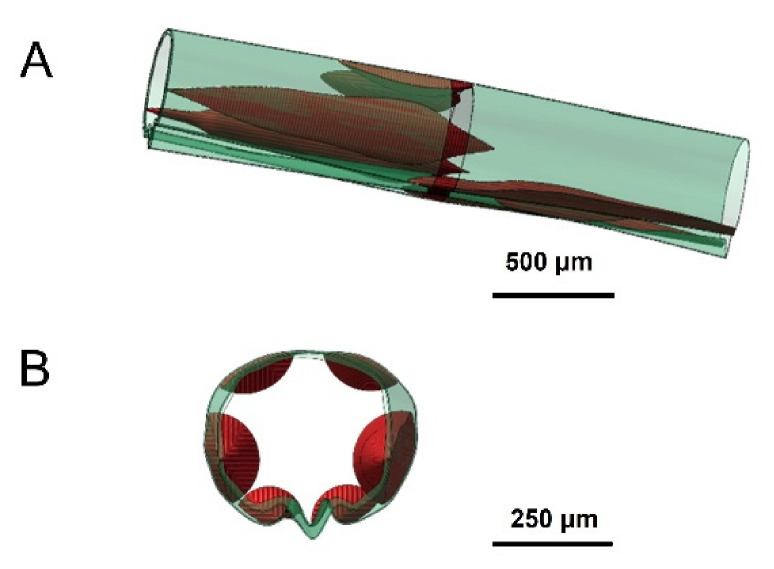
Geometric model of male *Ischnura elegans* abdominal segments VI to VII used for FEA. (**A**) Lateral and (**B**) posterior views, with the exoskeleton, muscles, and intersegmental membrane color-coded in green, red, and gray, respectively.

Based on the morphological characteristics of abdominal segments VI–VII of male *I. elegans*, a finite element model incorporating the chitinous exoskeleton, muscles, and intersegmental membrane was established (Figure 6A,B). The model primarily corresponds to the region from the distal half of segment VI to the proximal half of segment VII. The chitinous exoskeleton exhibits a slender cylindrical geometry (Figure 6A). The dorsal longitudinal muscles are mainly attached to the tergum, while the ventral lateral muscles are primarily attached to the lateral and ventral surfaces of the sternum (Figure 6B). The location of maximum muscle thickness, deformation trends, and the ratios of muscle thickness to segment diameter and length were all based on the measurements described in Section 3.2. The model was uniformly scaled up by a factor of two relative to the prototype size. While preserving the structural topology, articulation relationships, and mechanical force transmission pathways unchanged, this scaling improved mesh quality, solution stability, and the readability of the resulting contour plots, and also provided a more suitable reference for the subsequent design of the mechanical model.

**Figure 7:**
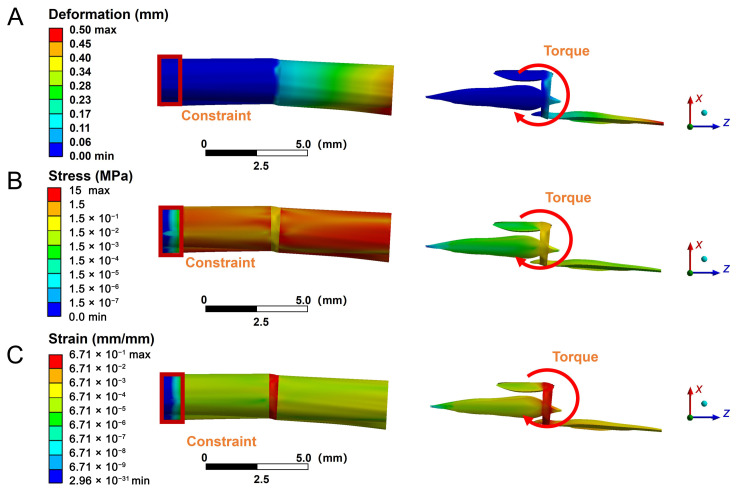
FEA results of abdominal segments VI–VII in male *I. elegans*. (**A**–**C**) Mechanical response contours; the left column shows the exoskeleton with intersegmental membrane, and the right column shows the musculature with intersegmental membrane: (**A**) displacement, (**B**) stress, and (**C**) strain distributions. The red box in the figure represents the range of constraints, and the arc arrow represents the direction of the torque.

Driven by equivalent muscle forces, the displacement field distribution generated by the composite structure of muscles, intersegmental membrane, and body wall in the biomimetic simulation was consistent with that of the biological prototype (Section 3.1). Specifically, displacement gradually increased along the longitudinal axis of the abdomen from the proximal constrained end to the distal free end, and the maximum terminal displacement in both cases was approximately 0.51 mm. These results indicate that the numerical model successfully reproduces the kinematic behavior of the biological prototype and that the combination of these three structures serves as the key structural basis for generating stable and continuous bending deformation (Figure 7A).

**Figure 8:**
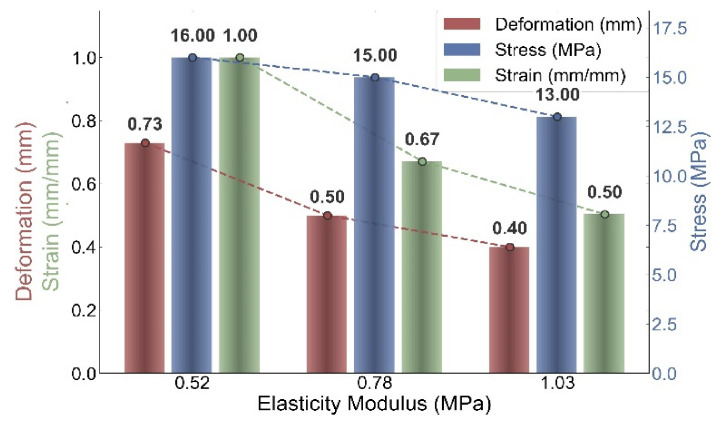
Sensitivity analysis of muscle elastic modulus. The chart illustrates the changes in maximum deformation (red), equivalent stress (blue), and equivalent strain (green) across three modulus values (0.52, 0.78, and 1.03 MPa), with dashed lines indicating the overall decreasing trends.

Based on these results, the sensitivity analysis reveals that the muscle elastic modulus exerts a significant regulatory effect on the magnitude of the abdominal bending response (Figure 8). As the muscle elastic modulus increases from 0.52 MPa to 1.03 MPa, the simulation output metrics exhibit a consistent, monotonic decline. Specifically, when the modulus rises from the baseline value of 0.78 MPa to the upper bound of 1.03 MPa, the maximum cumulative deformation decreases from 0.50 mm to 0.40 mm, the maximum equivalent strain drops from 0.67 to 0.50, and the maximum equivalent stress is reduced from 15.00 MPa to 13.00 MPa. Conversely, lowering the modulus to 0.52 MPa yields the highest structural responses, reaching a deformation of 0.73 mm, a strain of 1.00, and a stress of 16.00 MPa. This global trend demonstrates that the muscle elastic modulus acts as a critical tuning parameter that simultaneously governs the amplitude of structural deformation, strain accumulation, and internal stress distribution.

Comparative stress analysis revealed that high-stress regions were primarily concentrated on the intersegmental connection bands and muscle attachment sites, with a maximum equivalent stress of approximately 1 MPa, while most areas of the abdomen exhibited relatively low stress levels (Figure 7B). This demonstrates a mechanical characteristic of localized load concentration combined with globally stable load bearing. Plastic strain analysis showed that plastic strain in the exoskeleton was concentrated in the intersegmental membrane region, whereas plastic strain in the muscular system was concentrated at the muscle attachment interfaces (Figure 7C). Both contributed to the overall bending deformation through localized large deformation: the exoskeleton relied on elastic extension of the intersegmental membrane, while the muscular system depended on coordinated deformation at the muscle-integument attachment sites. This strain distribution provides critical insights for understanding the biological load-bearing mechanism and guiding subsequent bionic structural design.

The deformation distribution of the muscle tissue was generally consistent with the overall deformation trend of the abdominal segments but exhibited a smoother variation, with no obvious local collapse or unstable regions (Figure 7A). This suggests that abdominal bending does not rely on abrupt folding at a single point but is instead achieved through coordinated interaction between the muscle tissue and the abdominal body wall, producing smooth and continuous overall deflection. Further stress analysis indicated that high-stress regions were mainly concentrated at the muscle attachment sites and adjacent body wall connection bands, while most of the abdominal walls experienced low stress levels (Figure 7B). This implies that loads generated by active muscle contraction are first concentrated in local connection regions, then transmitted through the abdominal wall and converted into overall bending. No large-scale abnormal stress concentration was observed in the model, confirming that the structure of abdominal segments VI–VII maintains good mechanical stability under large-deflection conditions.

Therefore, we propose that the composite pattern of muscles, intersegmental membrane, and body wall in damselfly, including the material properties, morphological characteristics, and connection modes of these three tissue types, provides a reliable structural basis for achieving vector bending and holds potential value for application in the mechanical design of vectoring nozzles.

### 3.4. Damselfly-Abdomen-Inspired Nozzle Enables Stable Large-Angle Bending with Continuous Flow

The results of the multi-body dynamics simulation show that the biomimetic nozzle, constructed based on the composite structure model of the damselfly’s body wall, muscles and inter-somite membranes, achieved stable, continuous and large-angle bending (61.20° within 8 s) while maintaining the integrity of the flow channel. The bending process was smooth, without local instability, and the kinematic performance exhibited good stability without oscillations or reverse fluctuations.

**Figure 9:**
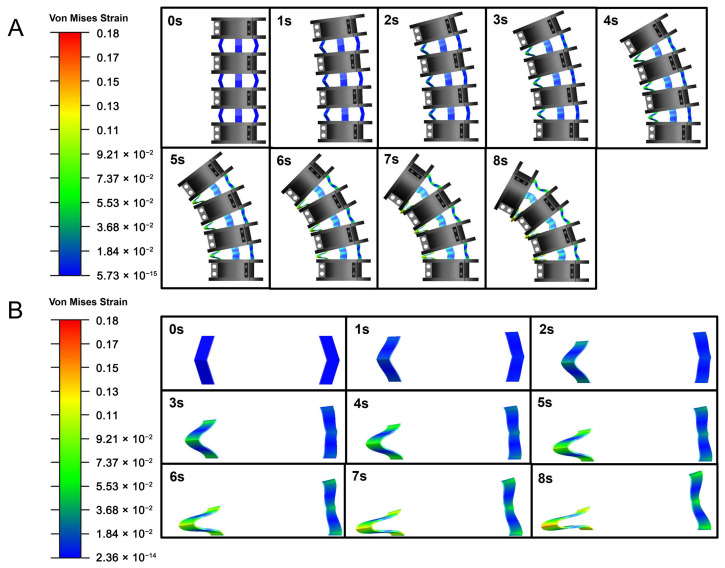
Bending process and strain distribution in the damselfly-inspired jet nozzle. (**A**) Time-series showing the overall nozzle bending and corresponding strain distribution. (**B**) Deformation and strain evolution in the inner and outer bending beams of the first layer over time. The results indicate that during bending, the outer beam is under tension while the inner beam is under compression. As bending progresses, the extent of tension and compression increases, with the peak strain reaching approximately 0.18. As the overall deflection of the nozzle increases, the strained region of the bending beams expands, with high-strain zones primarily concentrated in the central and end sections of the inner bending beams in each layer.

As shown in Figure 9A, at 0 s, the entire nozzle remains in a vertical state, and the axes of all segments are aligned. The curved beam does not show significant deformation. At 1 s, the first three segments show slight bending. Between 2 and 3 s, the bending degree becomes more obvious, and the centerline of the nozzle changes from a straight line to a continuous smooth curve. From 4 to 6 s, the bending amplitude increases significantly, and the differences in the postures of each segment gradually expand. From 7 to 8 s, the nozzle enters a large-bending state, forming a continuous, stable, and clearly directional bending shape. Throughout the process, the nozzle achieves a bending angle of 61.20° within 8 s. Although the overall deflection amplitude is large, the nozzle flow channel remains continuous, without any segment misalignment, breakage, or local instability, indicating that this biomimetic multi-segment structure can still maintain good geometric integrity under large deflection conditions. As shown in Figure 9B, as the deflection amplitude increases, the strain area of the curved beam gradually expands, and the high strain values are mainly concentrated in the middle and both ends of the inner curved beam. During bending, the outer beam is stretched, and the inner beam is compressed; as the bending degree increases, the stretching and compression degrees become more obvious. In the final bending state, each bending beam undergoes a certain degree of deformation, but there is no large-scale high-strain concentration phenomenon, indicating that the material selection and quantity configuration are suitable for multi-segment coupled motion.

**Figure 10:**
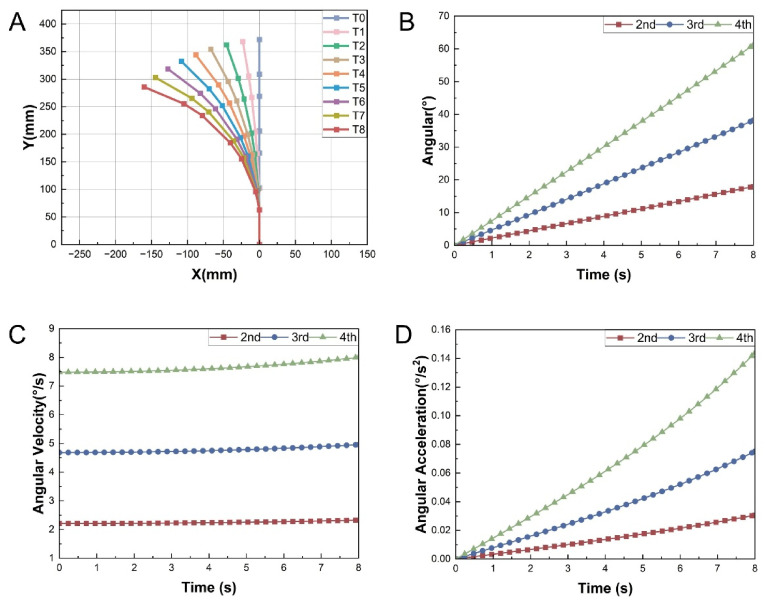
The motion trajectories and kinematic curves of the connection points of each section of the damselfly-like jet nozzle. (**A**) Trajectory diagram of the connection points of each section of the jet nozzle. (**B**) Curve of deflection angle. (**C**) Curve of deflection angular velocity. (**D**) Curve of deflection angular acceleration.

Figure 10 shows the motion trajectories and kinematic results of the connection points of each section of the dragonfly-like nozzle. As shown in Figure 10A, each section of the nozzle moves along continuous and smooth curves during the deflection process. The overall shape is highly consistent with the curved trajectory of the abdominal IV–VII segments of the damselfly during the formation of the mating wheel in Figure 4, indicating that this biomimetic nozzle can well reproduce the continuous and compliant deflection characteristics of the biological prototype. As shown in Figure 10B, the deflection angles of the second, third, and fourth sections of the nozzle increase continuously over time, with the maximum angle of the fourth section being 61.20°, the third section being 38.17°, and the second section being the smallest at 17.99°. This pattern corresponds to the result in Figure 4 that the displacements of the abdominal VII and VI segments are significantly higher than those of the anterior body segments. This is because the upper section is less restricted, and the bending of the lower section will have a cumulative and additive effect on the deflection of the upper section, resulting in a larger deflection angle for the upper section. As shown in Figure 10C,D, the deflection angular velocity and angular acceleration of the nozzle increase monotonically over time, and the values of the upper section are always greater than those of the lower section. From the angular velocity curve, the angular velocities of the second, third, and fourth sections change relatively smoothly over time, showing only a slight upward trend within the 0–8 s interval, without obvious oscillations, jumps, or reverse fluctuations. This indicates that the nozzle bending process has good motion stability.

In summary, the biomimetic nozzle constructed based on the composite structural pattern of the damselfly’s body wall, muscles, and intersegmental membrane successfully reproduces the synergistic mechanism observed in the biological prototype. Under large-deflection conditions, the nozzle exhibits excellent geometric integrity and kinematic stability, validating the feasibility of translating the biological abdominal bending mechanism into the engineering design of vectoring nozzles.

### 3.5. Aerodynamic Performance and Thrust Vectoring Verification

**Figure 11:**
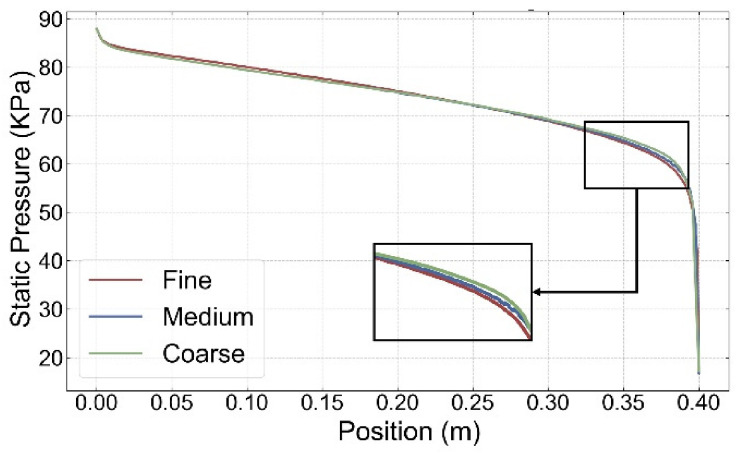
Grid independence verification. The plot compares the static pressure distributions along the nozzle centerline obtained from coarse, medium, and fine mesh configurations, with the inset highlighting the structural convergence at the exit zone.

**Figure 12:**
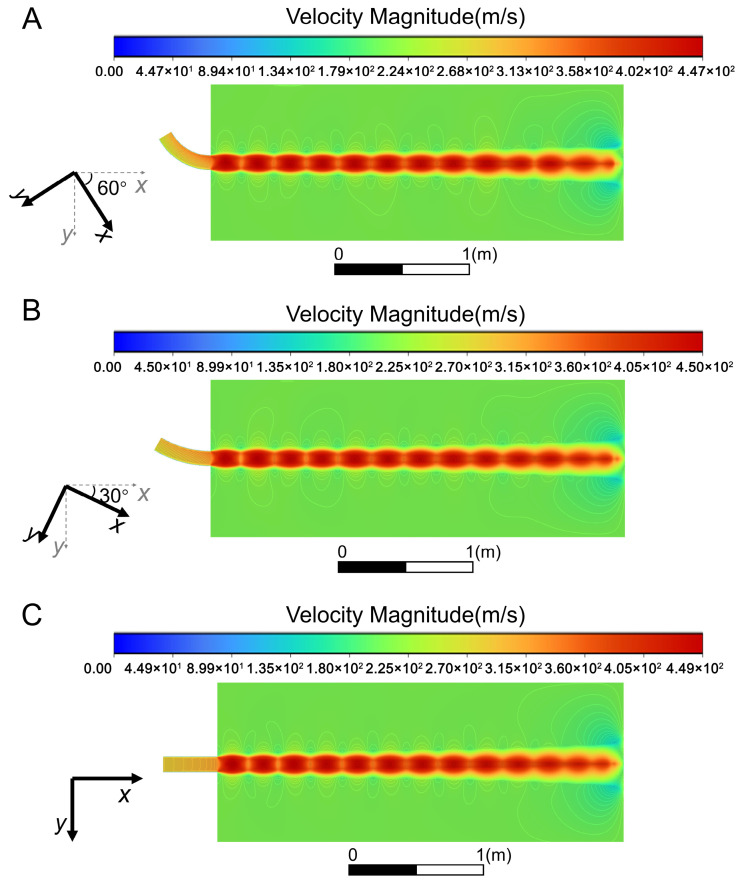
Velocity magnitude contours under different vectoring angles. The subplots display for (**A**) 60° upward deflection, (**B**) 30° upward deflection, and (**C**) the initial 0° state.

**Figure 13:**
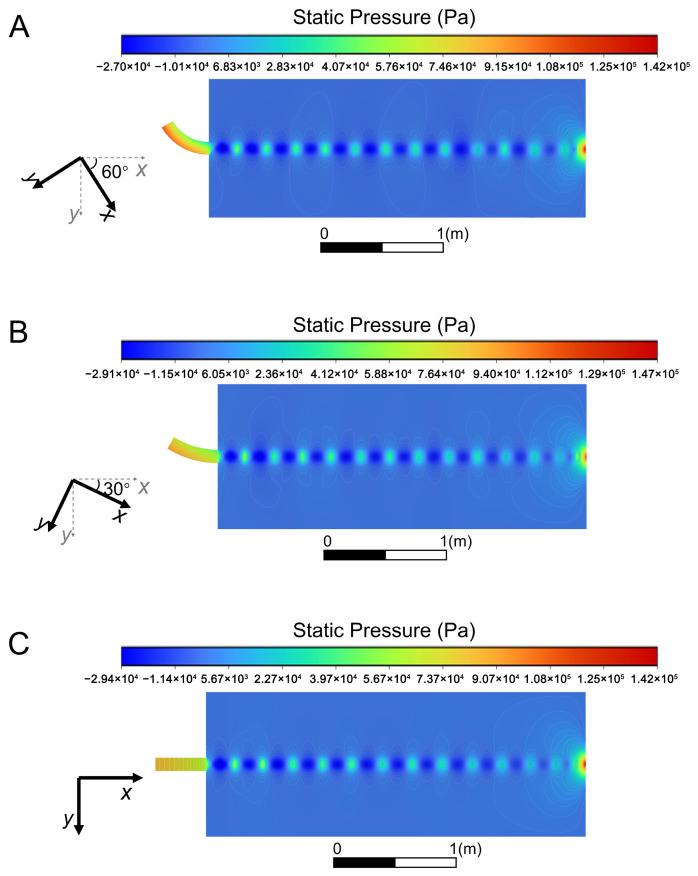
Static pressure contours under different vectoring angles. The subplots display for (**A**) 60° upward deflection, (**B**) 30° upward deflection, and (**C**) the initial 0° state.

To ensure the credibility of the numerical simulations, a grid independence study was initially conducted using three mesh density levels. As illustrated in Figure 11, the static pressure distributions along the nozzle centerline under coarse, medium, and fine meshes present excellent agreement, with the zoomed-in view showing that the deviation between the medium and fine meshes is negligible. Consequently, the fine-mesh model was selected to evaluate the fluid dynamic viability and thrust vectoring functionality of the bionic configuration under three typical operating postures, specifically the initial state (0°), 30° upward deflection, and 60° upward deflection. The resulting velocity magnitude and static pressure contours within and downstream of the nozzle are presented in Figure 12 and Figure 13.

Under all operating conditions, the jet exhibits a typical subsonic compressible flow structure (Figure 12 and Figure 13). The jet axis coincides exactly with the geometric angle of the nozzle exit, with no significant axis deviation or asymmetric divergence observed. The static pressure distribution shows a strict negative correlation with the velocity distribution, with alternating high-pressure/low-speed and low-pressure/high-speed regions, conforming to the fundamental fluid dynamics of subsonic compressible jets. These results demonstrate that the proposed vectoring nozzle possesses excellent jet directivity and precise vectoring capability across the 0–60° deflection range.

## 4. Discussion

This study systematically investigates the abdominal bending mechanism of *I. elegans* and applies its structural principles to a novel multi-segment vectoring nozzle configuration. Multibody dynamics and CFD simulations demonstrate that the bionic nozzle executes a stable, continuous deflection of 61.20° within 8 s under subsonic conditions, maintaining excellent flow-channel integrity and wall-attachment—a significant increase over traditional single-pivot mechanical or fluidic counter-flow nozzles, which are typically restricted to vector angles around 20° [4,7]. This aerodynamic and kinematic viability establishes a solid technical foundation for translating the biological distributed actuation into engineering hardware. It must be stressed, however, that these results alone do not yet confirm the engineering applicability of the proposed configuration. The present work is primarily a conceptual exploration of a new distributed-actuation paradigm; the simulations are not yet sufficient to support a direct, quantitative comparison with key performance metrics of existing vectoring nozzles, such as thrust efficiency, dynamic response bandwidth, or weight penalty. Moreover, the assumptions and simplifications underlying the current analyses leave open the possibility that the configuration may prove impractical or unreliable under real-world operating conditions.

In the proposed configuration, parallel muscle fibers are simplified into a multi-bundle cable-driven system housed within segmented spacer discs. Proximal hydraulic or linear actuators centralize power, enabling precise wire control without adding distal weight or inertia. The system can replicate damselfly bending via constant curvature or Cosserat beam models. Finite element analysis shows that muscle elastic modulus acts as the key parameter governing abdominal deformation and stress distribution. Consequently, in hardware, traction cable properties critically regulate vectoring performance: stiffness, compliance, and nonlinear elasticity directly affect force transmission from actuators to discs [20,42]. Selecting optimal cables (e.g., high-tensile braided steel or carbon fiber) is therefore essential to control strain concentration and ensure predictable, high-precision deflection curves.

Despite these design considerations, the physical integration of the cable-driven mechanism must be rigorously baselined for practical implementation. A notable risk arises when translating the biologically validated bending pattern into control inputs for a physical configuration [43]. The use of AI-enhanced frame interpolation (RIFE) in lieu of direct high-speed imaging can systematically underestimate peak displacement and velocity, as smoothing artifacts attenuate true instantaneous magnitudes during rapid acceleration [44]. If the cable-driven configuration is dimensioned solely around these attenuated motion profiles, it may encounter transient control lag and structural overload when commanded to execute the original, unattenuated trajectory. To maintain an adequate stability margin, this translation step necessitates conservative structural safety factors, increased actuator bandwidth, and elevated peak traction reserves [45,46].

The primary technical bottlenecks governing long-term operational reliability lie in the simplified numerical assumptions of the current investigation. Mechanically, the ideal frictionless joint contacts overlook cable friction, routing backlash, and component wear [47]. High-frequency friction within the cable conduits would inevitably induce mechanical hysteresis, leading to a prominent control dead-band and severe input–output asymmetry during rapid transient vectoring [42]. Simultaneously, mechanical backlash between the interlocking spacer discs would compromise vectoring precision and accelerate contact wear [48,49]. Aerodynamically, the two-dimensional simplification neglects three-dimensional flow expansion, secondary tip vortices, and pressure losses across the structural joints [50,51]. Furthermore, under real hot-gas exhaust propulsion, severe thermal loading creates massive thermal gradients across the segmented walls, inducing substantial thermal resistance and localized expansion stress [24,52]. When combined with intense acoustic vibrations and cyclic vectoring, this thermal load introduces a critical risk of structural fatigue at the interlocking interfaces [53]. Because these coupled multiphysics phenomena define the critical path for technology translation, future research must transition to comprehensive three-dimensional aerothermal–structural coupled analyses, while evaluating advanced high-temperature materials such as nickel-based superalloy corrugated seals and ceramic matrix composite flexible beams to validate physical engine prototypes [54,55].

## 5. Conclusions

In this study, a novel multi-segmented thrust vectoring nozzle configuration was developed by successfully translating the high-amplitude abdominal bending mechanics of the male damselfly *I. elegans* during mating wheel formation. Kinematic and morphological characterizations confirm that segments VI and VII serve as the critical structural nodes, while multibody dynamics and computational fluid dynamics simulations demonstrate that the bio-inspired nozzle executes a stable, continuous deflection of 61.20° within 8 s under subsonic conditions. By distributing the total deformation across sequential sub-joints, this configuration effectively smooths the inner flow path and suppresses boundary layer separation. Future research directions will transition from these simplified numerical environments to comprehensive three-dimensional aerothermal–structural coupled analyses to fully quantify internal pressure losses and thermal resistance under realistic propulsion conditions. Additionally, upcoming development phases will prioritize the integration of advanced high-temperature smart materials, such as nickel-based superalloy corrugated seals and ceramic matrix composite flexible beams, alongside physical prototype testing to evaluate and compensate for cable friction and joint backlash.

## Data Availability

The data presented in this study are openly available in Mendeley Data at: https://data.mendeley.com/datasets/df2h2dxw6c/1, accessed on 31 May 2026.

## Abbreviations

The following abbreviations are used in this manuscript:

TVC	thrust vector control
SEM	scanning electron microscopy
CLSM	confocal laser scanning microscopy
FEA	Finite Element Analysis
ADAMS	Automatic Dynamic Analysis of Mechanical Systems
3D	Three-dimensional
MNF	Modal Neutral File
CFD	Computational fluid dynamics
RIFE	Real-time Intermediate Flow Estimation
